# Aspirin for Venous Ulcers: Randomised Trial (AVURT): study protocol for a randomised controlled trial

**DOI:** 10.1186/s13063-015-1039-9

**Published:** 2015-11-10

**Authors:** Helen Tilbrook, Rachael O. Forsythe, Debbie Rolfe, Laura Clark, Martin Bland, Hannah Buckley, Ian Chetter, Liz Cook, Jo Dumville, Rhian Gabe, Keith Harding, Alison Layton, Ellie Lindsay, Catriona McDaid, Christine Moffatt, Ceri Phillips, Gerard Stansby, Peter Vowden, Laurie Williams, David Torgerson, Robert J. Hinchliffe

**Affiliations:** Department of Health Sciences, York Trials Unit, University of York, York, UK; St George’s Vascular Institute, St George’s Healthcare NHS Trust, Blackshaw Road, London, SW17 0QT UK; St George’s University of London, London, UK; Hull York Medical School, University of Hull, Kingston upon Hull, UK; School of Nursing, Midwifery and Social Work, University of Manchester, Manchester, UK; Wound Healing Research Unit, Cardiff University, Cardiff, UK; Harrogate and District NHS Foundation Trust, London, UK; The Lindsay Leg Club Foundation, London, UK; School of Health Sciences, University of Nottingham, Nottingham, UK; Swansea Centre for Health Economics, Swansea University, Swansea, UK; School of Surgical and Reproductive Sciences, Newcastle University, Newcastle upon Tyne, UK; University of Bradford, Bradford, UK

**Keywords:** Leg ulcer, Venous ulcer, Wound healing, Aspirin, Compression therapy

## Abstract

**Background:**

Venous leg ulcers (VLUs) are the commonest cause of leg ulceration, affecting 1 in 100 adults. There is a significant health burden associated with VLUs – it is estimated that the cost of treatment for 1 ulcer is up to £1300 per year in the NHS. The mainstay of treatment is with graduated compression bandaging; however, treatment is often prolonged and up to one quarter of venous leg ulcers do not heal despite standard care. Two previous trials have suggested that low-dose aspirin, as an adjunct to standard care, may hasten healing, but these trials were small and of poor quality. Aspirin is an inexpensive, widely used medication but its safety and efficacy in the treatment of VLUs remains to be established.

**Methods/Design:**

AVURT is a phase II randomised double blind, parallel-group, placebo-controlled efficacy trial. The primary objective is to examine whether aspirin, in addition to standard care, is effective in patients with chronic VLUs (i.e. over 6 weeks in duration or a history of VLU). Secondary objectives include feasibility and safety of aspirin in this population. A target of 100 participants, identified from community leg ulcer clinics and hospital clinics, will be randomised to receive either 300 mg of aspirin once daily or placebo. All participants will receive standard care with compression therapy. The primary outcome will be time to healing of the reference ulcer. Follow-up will occur for a maximum of 27 weeks. The primary analysis will use a Cox proportional hazards model to compare time to healing using the principles of intention-to-treat. Secondary outcomes will include ulcer size, pain evaluation, compliance and adverse events.

**Discussion:**

The AVURT trial will investigate the efficacy and safety of aspirin as a treatment for VLU and will inform on the feasibility of proceeding to a larger phase III study. This study will address the paucity of information currently available regarding aspirin therapy to treat VLU.

**Trial registration:**

The study is registered on a public database with clinicaltrials.gov (NCT02333123; registered on 5 November 2014).

**Electronic supplementary material:**

The online version of this article (doi:10.1186/s13063-015-1039-9) contains supplementary material, which is available to authorized users.

## Background

Venous leg ulcers (VLUs) are wounds of the lower limb caused by a diseased venous system, typically occurring in the gaiter area of the leg. VLUs represent the most common cause of leg ulceration, with a lifetime prevalence of 1–3 % in UK adults and accounting for around 85 % of all lower limb ulcers [1].

Many VLUs take over 6 months to heal; one large study demonstrated a median time to ulcer healing of 99 days with 2-layer compression therapy [2]. In addition, more than a quarter fail to heal completely [3] and the 12-month recurrence rate of healed VLUs may be up to 28 % [4, 5]. Patients with longstanding, large ulcers, or who have a prior history of ulceration, are particularly resistant to healing [6, 7]. VLUs impair quality of life (QoL); they are open wounds, which can be large, are often painful, frequently become infected and leak exudate. Compression bandaging is an effective treatment [8] but requires the use of sometimes bulky bandages alongside the need for regular clinic visits. Health-related QoL is decreased in patients with VLUs, which can incur significant psychological morbidity [9], and successful treatment has been shown to significantly improve QoL [10]. VLUs represent a significant health economic burden, costing up to £1300 to treat 1 VLU episode for a year in the UK [11]. There is, therefore, an unmet need for a more cost-effective and clinically effective treatment for VLUs.

### Pathophysiology of venous leg ulcers

In a healthy individual, flow of venous blood back to the heart occurs via the superficial venous system through the deep venous system, using the calf muscle pump and the venous valves to facilitate this flow against gravity. Resting hydrostatic venous pressure in the lower limb is 80 mmHg in the standing position, with no pressure gradient. When exercising, pressure in the deep venous system exceeds 80 mmHg, due to contraction of the calf muscles, forcing blood flow towards the heart. Valves in the superficial and perforator venous systems close to prevent retrograde flow. When the leg muscles relax again, pressure in the deep system falls below 80 mmHg, allowing blood to flow from the superficial system to the deep system through patent valves. Any dysfunction along this pathway may contribute to the development of venous ulceration.

VLUs most commonly result from impaired venous return due to calf muscle pump failure, usually as a result of obstruction or valve dysfunction in the superficial, deep or perforator venous system in the leg (primary venous disease). VLUs may also occur following a deep vein thrombosis or trauma (secondary venous disease). Other important factors include obesity and immobility.

Pathological maladaptation underlying VLUs include structural changes in vessel walls such as intimal hyperplasia, increased collagen content in areas of hypertrophy, as well as reduced smooth muscle cells and extracellular matrix [12]. These changes are likely triggered by inflammation and contribute to loss of venous tone and, ultimately, venous reflux and hypertension. These structural changes are also accompanied by cellular changes in the wound and wound bed – increased proteolytic activity, platelet aggregation and infiltration of leucocytes into the dermis, causing dermal fibrosis and leading to cutaneous changes such as lipodermatosclerosis, haemosiderin deposition and ulceration. Haemodynamic changes resulting from venous hypertension also affect the microcirculation, promoting interstitial oedema and capillary leakage. This combination of inflammatory activities may cause the VLU to heal slowly, or not at all. Targeting and reversing these pathophysiological pathways is the focus of adjunctive drug treatment.

### Current treatment of venous leg ulcers

Careful and regular clinical assessment should be the first step in the management of venous ulceration and should ideally be performed in a specialised venous ulcer clinic. All patients should have a venous Duplex scan to assess for treatable venous disease. Ulcer area and characteristics should be monitored over time, as the changing nature of an ulcer (depth, area, base, ulcer edge) can indicate progression of disease or healing. Bacteriological swabs and antibiotics should only be used in cases of proven clinical infection and a biopsy may be considered in cases of atypical or non-healing ulceration. Simple dressings, meticulous wound care and judicious sharp debridement should be undertaken by experienced practitioners. All patients with VLUs should have ankle brachial pressure index (ABPI) performed prior to the instigation of treatment to exclude arterial disease and should have cardiovascular risk factors addressed in the presence of an abnormal ABPI, in addition to referral to a vascular surgeon. Compression therapy should be instigated and undertaken by an appropriately trained professional. According to Scottish Intercollegiate Guidelines Network (SIGN) guidelines, patients with a chronic non-healing VLU and concomitant superficial venous reflux should be referred for consideration for surgery to prevent recurrence [13].

### Compression therapy

The standard treatment of VLUs is multi-layered compression bandaging (aiming for a pressure of 40 mmHg at the ankle [14] with the aim to reduce venous hypertension, improve calf muscle function and create a wound environment that encourages healing whilst reducing tissue maceration and excessive oedema and moisture. Compression is recommended as first-line treatment for VLUs in major UK guidelines [13].

The ‘gold standard’ is four-layer multi-component compression therapy [15]; however, this is often considered unsightly and uncomfortable due to the bulky nature of the bandages, and may restrict movement at the ankle, making it difficult to wear shoes. In addition, poor application technique may reduce the effectiveness of compression and the negative physical and social impact of compression stockings may lead to ambivalence about their effectiveness and subsequently lead to non-compliance [16]. Two-layer compression stockings are an alternative to four-layer bandaging and a recent randomised trial has demonstrated a reduction in ulcer recurrence with the two-layer approach [2, 17]. Various single-layer hosiery are also available; however, these do not meet the 40-mmHg targeted compression pressure.

### Topical therapy

Topical therapies have been used for VLUs (including silver-containing antibiotics, zinc oxide and other topical antimicrobials or impregnated dressings) although there is no reliable evidence to suggest that complex wound dressings are better than simple non-adherent dressings [18]. Topical local anaesthetic creams may help bring symptomatic relief when the ulcers are painful.

### Adjunctive drug treatment

Various drug adjuncts to compression have also been investigated, with a recent Cochrane Review demonstrating that pentoxyfilline (a vasodilator that decreases blood viscosity, modifies leucocyte activity and has some anti-platelet effects) is effective in improving wound healing when used with, and possibly without, four-layer compression [19]. However, vasodilators such as pentoxyfilline are not routinely prescribed in the National Health Service (NHS) and may produce intolerable adverse events (AEs), including potentially life-threatening side effects such as haematemesis, gastrointestinal haemorrhage and thrombocytopenia [20]. There is insufficient evidence to recommend the use of other adjunctive drugs, including venoactive drugs that increase venous tone via mechanisms that remain largely unclear.

### Aspirin

Aspirin is a cyclooxygenase inhibitor that irreversibly reduces prostaglandin-2 and thromboxane A2, which are involved in inflammation and platelet aggregation [21]. It is inexpensive, widely used and readily available. The mechanism by which aspirin may hasten healing of VLUs is unclear but may be associated with a reduction of inflammation, or its effect on the microvascular circulation, including platelet activation. In one study investigating the haemostatic effects of aspirin in patients with VLU, the investigators demonstrated that participants were found to have increased levels of fibrinogen and shortened coagulation rate, when compared to age-matched and sex-matched controls and that treatment with aspirin caused prolongation of the coagulation rate, which increased the rate of ulcer healing [22].

There have been 2 small randomised trials to date that have investigated the use of aspirin (300 mg) in VLUs; however, the quality of evidence presented was low and more robust studies are required to confirm their findings. An additional file outlines the previous studies investigating aspirin in VLUs (see Additional file 1).

The first study was carried out in 1994 and demonstrated that 38 % more patients healed in the treatment group (aspirin plus compression) than in the control group (placebo plus compression) [23]; however, no patients healed within 4 months in the control group, which is surprising, given that the median time to healing with compression alone is around 3 months [15]. Although it provides some limited data about the potential use of aspirin therapy, the sample size of only 20 patients is insufficient to draw meaningful conclusions. In addition, patients were only followed-up for 4 months.

Over a decade later, a Spanish group conducted a small randomised pilot trial (*n* = 51 patients) of aspirin and compression, demonstrating that aspirin reduced the average time to healing but did not influence the rate of healing and had no effect on the rate of ulcer recurrence. In addition, after multivariate analysis was performed, aspirin was not demonstrated to be an independent predictor of healing [24] with only initial ulcer size at study entry remaining independently associated with rate of healing. Moreover, no information was presented regarding the placebo and there is uncertainty around the effect estimates. The quality of evidence that aspirin hastens healing of VLUs is, therefore, low and needs addressing through more robust studies.

In addition to the Aspirin for Venous Ulcers: Randomised Trial (AVURT), there are two ongoing randomised trials investigating the use of aspirin in VLU. ASPiVLU (ASPirin in Venous Leg Ulcer healing, ACTRN12614000293662) will investigate the use of 300 mg aspirin, in addition to standard 3-layer compression therapy, with the primary endpoint as the time to complete ulcer healing at or before 12 weeks from randomisation. Aspirin4VLU (Low Dose Aspirin for Venous Leg Ulceration, NCT02158806) will investigate 150 mg aspirin, in addition to routine care, on time to complete healing of the reference ulcer. In addition to the trials reporting individually, data from AVURT, ASPiVLU and Aspirin4VLU will be combined in order to carry out an individual patient data (IPD) meta-analysis. Any other relevant trials such as the two earlier trials will also be considered for inclusion.

### Other options: surgery and minimally invasive intervention

Varicose vein surgery for VLUs has not been shown to influence the time to VLU healing; however, it may decrease the rate and severity of recurrence [4, 25],. Minimally invasive techniques such as radiofrequency ablation, foam sclerotherapy and endovascular laser ablation have largely replaced traditional open surgical techniques in the treatment of varicose veins, where possible. There have been no large-scale randomised trials investigating the superiority of one technique over another when treating VLUs, although recent studies suggest some benefit from radiofrequency ablation to assist VLU healing [26]. However, a large multi-centre randomised trial is currently underway (EVRA – Early Venous Reflux Ablation ulcer trial), aiming to assess the influence of early endovenous treatment of superficial venous reflux in patients with VLUs, compared to standard compression therapy (NIHR HTA 11/129/197; ISRCTN02335796).

### Other options: cell-based therapy, skin grafts and acellular products

Research on novel treatments with cell-based therapy is currently in progress, with promising results from phase II and phase III trials investigating the use of allogenic cells, either applied topically or via injection onto areas of ulceration [27, 28] as well as growth factors [29]. However, such therapies are expensive, may be associated with significant side effects and are unlikely to become widely available in the near future. Acellular products, such as porcine mucosa, have been trialled to assist VLU healing, with promising results noted in one study [30]. A recent Cochrane Review of skin grafting for VLU (including autografts, allografts, xenografts and bioengineered artificial skin grafts) demonstrated that bilayer tissue-engineered skin replacement, used with compression, was the only skin grafting technique that may increase the rate of VLU healing [31], but data are very limited in this area.

### The need for AVURT – a randomised, placebo-controlled efficacy study

Whilst there have been two small trials to date that have investigated the use of aspirin in the treatment of VLUs, the quality of the evidence presented is low. Given the significant health burden represented by VLUs, and the challenges in treating the disease, there is a need to identify effective, inexpensive, safe and widely available treatments that patients may tolerate. The AVURT seeks to investigate the effect of aspirin on time to healing of VLUs, to examine safety issues in this cohort of patients and to inform on the feasibility of proceeding from a phase II trial to an efficacy and effectiveness (phase III) trial. If a simple, cheap and well-tolerated medication, such as aspirin, were to result in a reduction in time to healing, this would impact on patient management, resource use and the potential impact on the population is substantial, given that aspirin is widely available. Meta-analyses have demonstrated that low-dose aspirin increases the risk of major bleeding compared to placebo [32, 33]; however, the absolute increase is modest and there is no evidence that decreasing the dose will reduce the risks of side effects [34]. The study will also provide the opportunity to systematically review the safety profile of aspirin in this population of patients, as well as assess the generalisability of the medication by studying the number of patients with VLUs who are currently taking aspirin or other anti-platelet medications.

## Methods/Design

### Trial design

The AVURT is designed to inform the feasibility of a larger, confirmatory study of aspirin therapy for VLU. AVURT is a phase II randomised, double blind, parallel-group, placebo-controlled study to provide evidence regarding the efficacy and safety of aspirin (at a dose of 300 mg once daily), in addition to standard care in patients with chronic VLUs. A chronic VLU is defined as any break in the skin that has either: a) been present for more than 6 weeks, or b) occurred in a person with a history of venous leg ulceration. Ulcers will be considered venous if no other aetiology is clinically suspected. The ulcer must be venous in appearance (i.e. moist, shallow and irregular of appearance) and lie wholly or partially within the gaiter area of the leg. Potential participants will be identified from hospital outpatient clinics or community leg ulcer clinics, where they usually receive treatment for VLUs. An additional file shows the schematic of the AVURT trial design (see Additional file 2). All participants will continue to receive ‘standard care’ according to an evidence-based standardised approach to the management of VLUs, as per SIGN guidelines [13] with multi-component compression therapy aiming to deliver 40 mmHg of pressure at the ankle. The type of dressing used will be at the discretion of the healthcare professional managing the patient and will be documented in the participant case report form (CRF).

Aspirin will be provided as a 300-mg capsule identical in weight, colour and size to the matched placebo capsules. Placebo capsules will contain a lactose and magnesium stearate blend. Capsules will be packaged into child-resistant tamper evident bottles sufficient in size to hold 190 doses for the participant to complete 24 weeks treatment.

### Ethical approval

Full ethical approval has been granted by the National Research Ethics Service (NRES) East Midlands – Nottingham 2 research ethics committee (REC) (reference 14/EM/1305).

### Screening, eligibility and patient pathway

Screening will be conducted by research nurses, who will also identify potential participants, gain informed consent and conduct a baseline assessment. Patients will be recruited from hospital and community-based ulcer clinics, and through liaison with general practitioners (GPs), community nurses and hospital staff. Eligibility will be confirmed by a doctor. The participant will continue with regular (usually weekly or 2-weekly) visits to the usual place of ulcer care, where the research or treating nurse will assess the components involved in the study. An additional file shows a summary of AVURT assessments (see Additional file 3).

### Inclusion criteria

Inclusion criteria are:

- Patients with at least one chronic venous leg ulcer (if more than one ulcer, the largest ulcer will be chosen as the reference ulcer for the purposes of the trial)
- Ulcer area > 1 cm^2^
- ABPI ≥ 0.8 taken within the previous three months, *or*
- if the ABPI is incompressible, other forms of clinical assessment must exclude peripheral arterial disease (peripheral pulse examination, toe pressure, Duplex ultrasound, clinical judgement)
- Age over 18 years (no upper age limit)

## Informed consent

### Exclusion criteria

The exclusion criteria are:

- Unable to provide consent
- Unwilling to provide consent
- Foot ulcer (i.e. below the ankle)
- Leg ulcer of non-venous aetiology
- ABPI < 0.8 or, where ABPI is not compressible, peripheral arterial disease (PAD) cannot be excluded by other assessments
- Regular concomitant aspirin
- Previous intolerance or contraindication to aspirin use (according to prescriber’s clinical judgement)
- Prohibited medication: probenecid; oral anticoagulants including coumarins (warfarin, acenocoumarol) and phenindione; dabigatran; rivaroxaban; apixiban; heparin; clopidogrel; dipyridamole; sulfinpyrazone and iloprost
- Known lactose intolerance
- Pregnant/lactating women
- Male or pre-menopausal female participants of child-bearing potential^1^ unwilling to use an effective method of birth control (either hormonal in the form of the contraceptive pill or barrier method of birth control accompanied by the use of a proprietary spermicidal foam/gel or film; or agreement of true abstinence (i.e. withdrawal, calendar, ovulation, symptothermal and post ovulation are not acceptable methods) from time of consent is signed until 6 weeks after the last dose of Investigational Medicinal Product (IMP)
- Already participating in another study investigating leg ulcer therapy
- Previously been recruited into this trial
- Another reason that excludes them from participating within this trial (decision made according to the nurses’ or prescribers’ clinical judgment)

There will be no exceptions (waivers) to eligibility criteria. Participants will be considered eligible if they meet all of the inclusion criteria and none of the exclusion criteria mentioned above. Details of all screened patients, whether recruited or not, will be entered onto the sponsor screening log.

### Consent

The process of consent will be carried out in accordance with the Declaration of Helsinki. All patients will be fully informed about the nature of the research study and the chances of being randomised to either the trial drug (aspirin) or placebo. Written information will be provided to patients, who will have the opportunity to discuss the study with a member of the trial team prior to enrolment in the study. Patients will be aware that their decision to participate in the study is voluntary and that they are free to withdraw consent at any time with no effect on the standard treatment they receive. Written consent forms will be obtained from patients willing to participate in the study and will be retained by the investigator.

### Randomisation and blinding

Participants will be randomised on a 1:1 basis to receive either aspirin (300 mg) or placebo, in addition to standard care. The Research Pharmacy responsible for dispensing all trial medication (St George’s Hospital) will receive a randomisation schedule generated in advance by the IMP manufacturer, Sharp Clinical Services UK Ltd. (Sharp Clinical Services, UK) Stratification will be by ulcer size (≤5 cm^2^ or > 5 cm^2^).

Randomisation will be performed by the Research Pharmacy upon receipt of a valid prescription for a participant. Researchers, treating staff, clinicians and participants will be blind to treatment allocation. A 24-hour code-breaking service will be provided by the Research Pharmacy in case of requirement for emergency unblinding and participants will receive a study-specific 24-hour emergency contact card.

### Sample size calculation

This study aims to recruit 100 patients, which is sufficient to demonstrate whether there is evidence for efficacy of aspirin to treat VLUs, in line with previous similar trials [23, 24] and is also large enough to test the feasibility of study procedures such as recruitment.

The primary outcome is time to healing of the reference ulcer. Applying an assumed standard error for the hazard ratio (HR) of 0.105 following adjustment for log area and log duration of ulcer (as in VenUS IV) to the smaller sample size in this study implies that the standard error would be 0.22. A 95 % confidence interval (CI) for the log hazard ratio would thus be log(HR) ± 0.435. Hence, if the hazard ratio for this study were the same as that suggested by previous studies (around 1.5), the confidence interval would be (0.97, 2.31) which just includes 1.00. To further increase the power an IPD meta-analysis is proposed. As compliance and follow-up will be measured as part of the study there is no formal inflation for dropout.

An important secondary outcome is wound area. Assuming a standard deviation of 1.09 following log transformation as in (VenUS I) [15], 2 groups of 50 participants will render 80 % power to detect a difference of 0.62 on the natural log scale. This corresponds to a reduction of 46 % in ulcer area at follow-up. In the current study, there will be multiple measurements of wound area and so smaller differences should be detectable.

### Primary outcome

The primary outcome is time to ulcer healing, which will be defined as ‘completed epithelial healing in the absence of scab (eschar) with no dressing required’. This will take the form of survival time data for analysis. Time to healing will be measured in days from the date of randomisation until the first date that healing is recorded. If healing occurs before the end of the study, the participant will be followed for a further 2 weeks to confirm healing, in accordance with the Food and Drug Administration (FDA) and European Wound Management Association (EWMA) guidelines [35]. A digital photograph of the area will be taken at this point to confirm healing. For patients who have not healed, time from date of randomisation until they exit the trial, withdraw, are lost to follow-up or die will be used in the survival analysis – whichever occurs first.

### Secondary outcomes

Secondary outcomes are:

- Ulcer size (area) measured in cm^2^ using image analysis by SigmaScan, Systat Software Inc, San Jose, CA, USA and/or wound tracings
- Recurrence of reference ulcer
- Adverse events (AEs)
- Ulcer-related pain using a visual analogue scale
- Treatment compliance (capsule counting and nurse assessment of compression concordance)
- Resource use: number of wound consultations and types of dressings used

### Statistical analysis

Analyses will be in accordance with the principles of intention-to-treat. Analysis will be conducted in Stata® (Stata Corporation, College Station, TX, USA) or similar statistical software. Statistical significance will be assessed at the 2-sided 5 % level unless otherwise stated. Ninety-five percent CIs will be provided as appropriate. Statistical analyses will be detailed in an analysis plan that will be independently reviewed and agreed before data are analysed.

#### Primary outcome analyses

Time to ulcer healing will be presented by trial arm using a Kaplan-Meier plot and a log-rank survival comparison will be made. The median time to healing will be presented overall and by trial arm with corresponding 95 % CIs. The primary analysis will investigate differences between trial arms in relation to time to ulcer healing using a Cox proportional hazards regression model. Adjustments will be made for log transformed area and duration of the reference ulcer. The model will be tested for inclusion of shared centre frailty effects.

#### Secondary outcome analyses

Ulcer area will be transformed and investigated on the natural log scale through mixed models to see whether there are differences by trial arm.

The proportion of patients who are found to have a recurrence within the study period will be reported by trial arm. Time from healing to recurrence will be investigated in a similar fashion to the primary outcome should numbers be sufficient to allow.

AEs will be reported overall and by trial arm in terms of number of patients with at least one event and total number of events. Serious and non-serious events will be presented separately and according to whether they are thought to be related, or unrelated, to treatment. Differences in total numbers of events by trial arm will be compared using negative binomial regression adjusting for size and duration of ulcer.

Mean and median pain scores will be presented by trial arm and differences in pain scores between the allocated groups will be investigated using linear regression adjusted for baseline pain score.

Compliance will be reported in terms of proportion of patients completing the course of treatment up to healing or planned trial exit and compared between arms using a Chi-squared test and 95 % CIs.

Resource use will be presented using summary statistics in relation to the number of wound consultations per week and change to compression therapy or primary wound dressings.

### Treatment period and follow-up

After consent, participants will be screened to ensure eligibility. Prior to randomisation, baseline demographic details will be collected and a clinical assessment of the patient and wound performed. Following randomisation, participants will continue in the normal care pathway of weekly or 2-weekly clinical assessments at community ulcer clinics, hospital outpatient clinics or home visits and will not be required to attend any further visits for research purposes. All randomised participants will receive aspirin or placebo for 24 weeks and will be followed-up for 25 weeks following randomisation. If the reference ulcer is confirmed as healed during the follow-up period, then a photograph will be taken and the participant will continue to take the IMP or placebo for 2 further weeks. They will then be re-assessed (as per FDA and EWMA guidelines on wound healing) [35]. If the ulcer is confirmed as healed at this reassessment visit, then the date of ulcer healing will be recorded as the date that the ulcer was first assessed as healed. The participant will then be advised to stop taking the IMP or placebo. If a new ulcer occurs on the reference leg before the end of the study, then participants will be asked to inform the study team.

If the ulcer is assessed as ‘not healed’, then the participant will continue in the trial until the minimum period of follow-up (25 weeks) has elapsed providing confirmed healing does not occur before the end of the follow-up period. Both of these time-points (first healing judgement and confirmation of ulcer healing) will be recorded.

Participants will also be asked to provide a pain score using a visual analogue scale at baseline and 4–6 weeks after first dose of IMP. Weekly (or 2-weekly, if that is the participant’s usual interval of care) assessments will include: healing outcomes, treatment concordance with IMP and compression bandaging, AEs or side effects, change to concomitant medication, resource use (number of visits, types of dressings used and level of compression). Digital photographs, or leg ulcer tracings, will also be taken by the treating or research nurse.

### Safety reporting

Despite some apparent advantages of aspirin therapy in the treatment of VLUs, the risks associated with aspirin will carefully reported. Safety reporting during this trial is paramount and will be conducted in line with Healthy Technology Assessment (HTA) guidelines. Reportable safety events will include any of the following experienced by a participant during the trial: AE, adverse reaction, serious adverse event (SAE), serious adverse reaction (SAR), suspected unexpected SAR. All AEs will be recorded in the clinic notes, on the study CRF and reported to the sponsor via the sponsor AE log. SAEs and SARs will be notified to the sponsor immediately when the investigator becomes aware of the event (within 24 hours). The sponsor will inform the Medicines and Healthcare products Regulatory Agency (MHRA) and ethics committee, where appropriate. SAEs will be reported to the trial coordinator in the York Trials Unit via the sponsor and reviewed by the data monitoring committee.

All patients who develop unacceptable treatment toxicity which, in the investigator’s opinion, is attributable to the IMP or an SAE, will be withdrawn from the study treatment but follow-up will continue (where appropriate) to enable an intention-to-treat analysis. The side effects associated with aspirin are well-known to health professionals and no additional training will be required. These include, but are not limited to, gastrointestinal haemorrhage and gastrointestinal disturbance (including dyspepsia, ulceration).

In addition, AEs associated with leg ulceration or compression therapy will be recorded. Pregnancy and breastfeeding are exclusion criteria for the study; however, all patients of childbearing age will be advised to use barrier contraception during the duration of the study.

## Discussion/Summary

Chronic VLUs are a common medical problem associated with considerable morbidity. Current treatment (using graduated compression therapy) may not result in sustained wound healing; however, there is inadequate evidence of other effective alternatives, or adjuncts, to improve outcomes. Low-dose aspirin (in addition to standard compression therapy) may hasten healing; however, current evidence supporting its use is insufficient. This randomised trial will inform on whether low-dose aspirin is an effective, feasible and safe therapy for patients with chronic VLUs, in addition to standard compression therapy. This could go some way towards addressing the significant health burden associated with VLUs.

## Trial status

At the time of submission, the trial is open to recruitment. Collaborating centres include St George’s, University of London; University of York; University of Manchester; Bradford Teaching Hospitals NHS Foundation Trust; Harrogate and District NHS Foundation Trust; Hull and East Yorkshire Hospitals NHS Trust; University of Nottingham; Cardiff University; Newcastle University.

## Additional files

Additional file 1: Table S1. Previous randomised trials investigating aspirin in the treatment of VLU. (DOCX 15 kb) Additional file 2: Figure S1. Schematic of AVURT trial design. (PDF 88 kb) Additional file 3: Figure S2. Summary flow chart of AVURT assessments. (PDF 173 kb)

## Abbreviations

ABPI: ankle brachial pressure index
AE: adverse event
ASPiVLU: ASPirin in Venous Leg Ulcer healing
AVURT: Aspirin for Venous Ulcers: Randomised Trial
CI: confidence interval
CRF: case report form
EVRA: Early Venous Reflux Ablation ulcer trial
EWMA: European Wound Management Association
FDA: Food and Drug Administration
GP: general practitioner
HR: hazard ratio
HTA: Health Technology Assessment
IMP: Investigational Medicinal Product
IPD: individual patient data
MHRA: Medicines and Healthcare products Regulatory Agency
NHS: National Health Service
NIHR: National Institute for Health Research
NRES: National Research Ethics Service
QoL: quality of life
REC: research ethics committee
SAE: serious adverse event
SAR: serious adverse reaction
SIGN: Scottish Intercollegiate Guidelines Network
VLU: venous leg ulcer
PAD: peripheral artery disease

## Footnotes

1 Subjects are only considered not of child bearing potential if they are surgically sterile (i.e. they have undergone a hysterectomy, bilateral tubal ligation, or bilateral oophorectomy) or they are postmenopausal.
