# Supplementary material for: Aspirin for Venous Ulcers: Randomised Trial (AVURT): study protocol for a randomised controlled trial
Source: Trials. 2015 Nov 10;16:513. doi: 10.1186/s13063-015-1039-9 (PMC4641424; doi:10.1186/s13063-015-1039-9)
Supplement: Additional file 1: Table S1. — Previous randomised trials investigating aspirin in the treatment of VLU. (DOCX 15 kb) [file 13063_2015_1039_MOESM1_ESM.docx]

Table S1: Previous randomised trials investigating aspirin in the treatment of VLU

| **Author** | **Year** | ***n*** | **Type of study** | **Treatment group** | **Control group** | **Main results** |
| --- | --- | --- | --- | --- | --- | --- |
| Layton | 1994 | 20 | Double-blind randomised | Aspirin 300mg plus compression | Placebo plus compression | Ulcer healing within 4 months: 38% in treatment group vs 0% in control group (p<0.007).  Reduction in ulcer size: 52% in treatment group vs 26% in placebo group (p<0.007). |
| del Río Solá | 2012 | 51 | Double-blind randomised | Aspirin 300mg plus compression | Compression only | Complete healing: no difference between groups.  Time to healing: 12 weeks in treatment group vs 22 weeks in control group (p=0.04).  Ulcer recurrence: no difference between groups.  Initial area of injury was the only variable that influenced the rate of healing. |
