# Supplementary figures and images for: Aspirin for Venous Ulcers: Randomised Trial (AVURT): study protocol for a randomised controlled trial
Source: Trials. 2015 Nov 10;16:513. doi: 10.1186/s13063-015-1039-9 (PMC4641424; doi:10.1186/s13063-015-1039-9)

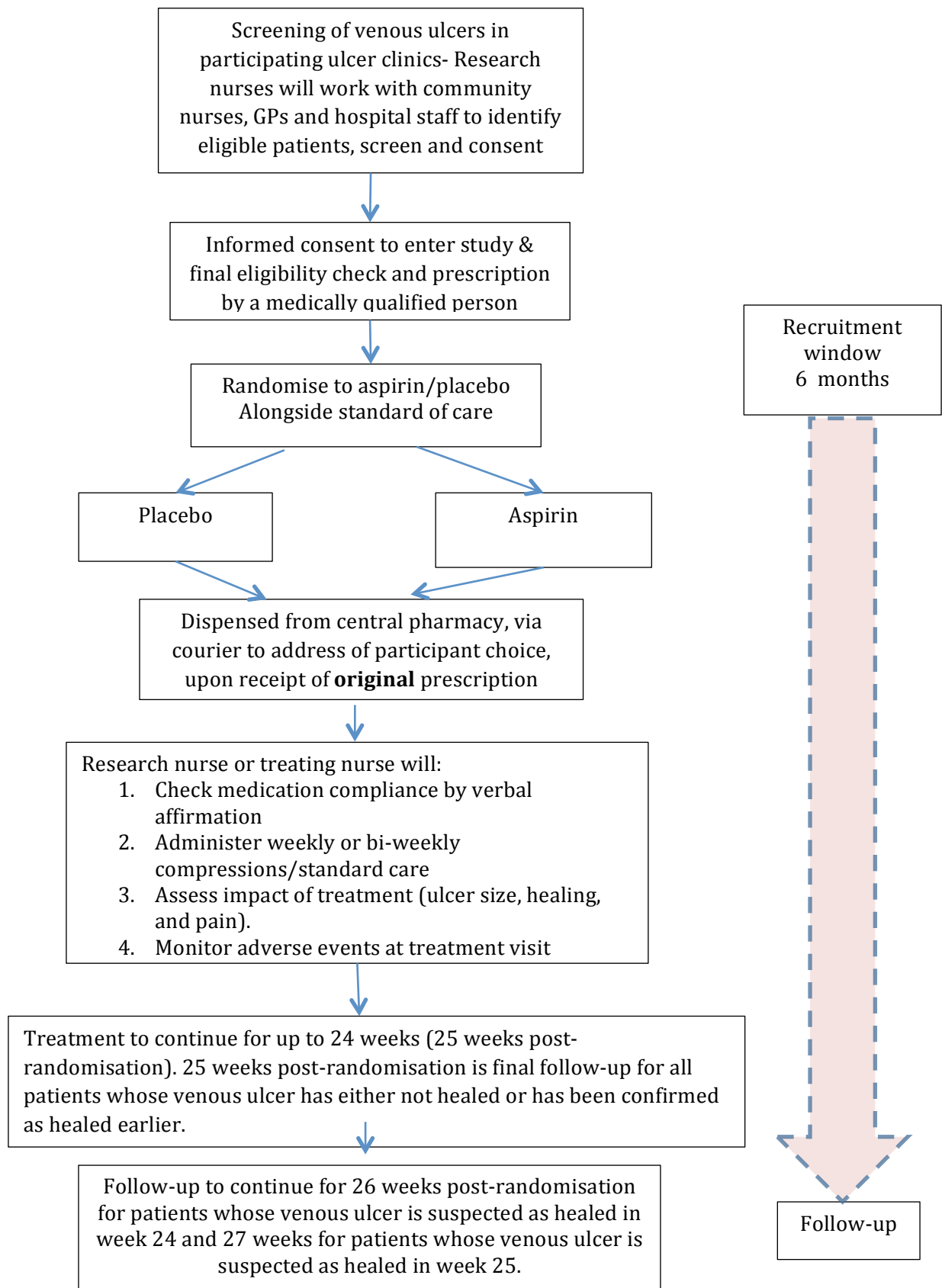

Supplement: Additional file 2: Figure S1. — Schematic of AVURT trial design. (PDF 88 kb) [file 13063_2015_1039_MOESM2_ESM.pdf]

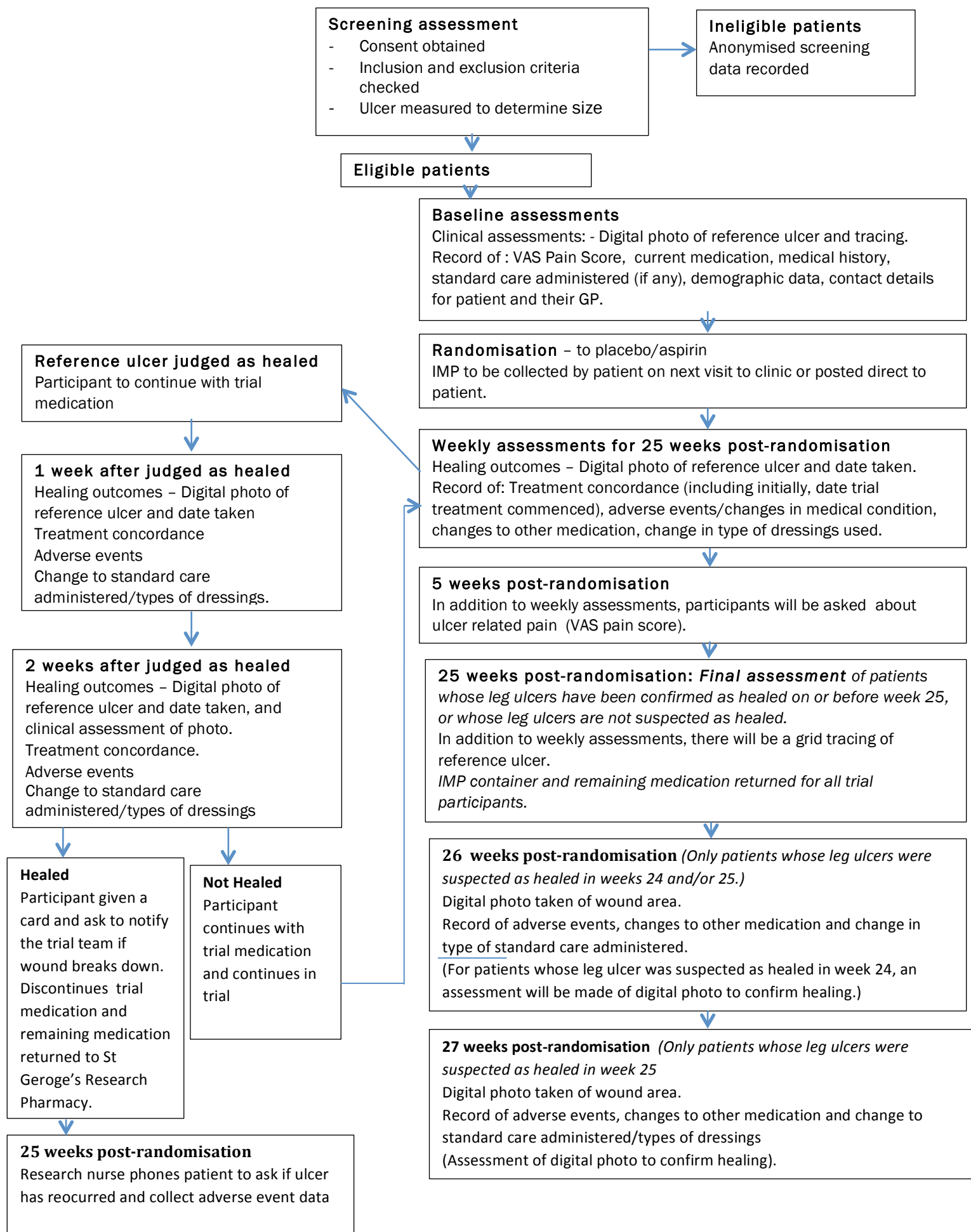

Supplement: Additional file 3: Figure S2. — Summary flow chart of AVURT assessments. (PDF 173 kb) [file 13063_2015_1039_MOESM3_ESM.pdf]
